# Enhancing cardiac disease prediction with explainable bidirectional LSTM

**DOI:** 10.1038/s41598-025-25071-8

**Published:** 2025-11-21

**Authors:** Swati Lipsa, Ranjan Kumar Dash, Subhra Debdas, Korhan Cengiz, Pankaj Kumar, Nitai Pal

**Affiliations:** 1 School of Computer Sciences, Odisha University of Technology and Research, Bhubaneswar, 751029 Odisha India; 2 School of Electrical Engineering, Kalinga Institute of Industrial Technology Deemed to be University, Bhubaneswar, 751024 Odisha India; 3 Department of IT, Istinye University, Istanbul, 751029 Turkey; 4 Department of Electrical and Electronics Engineering, Manipal Institute of Technology, Manipal Academy of Higher Education, Manipal, India; 5 Department of Electrical Engineering, Indian Institute of Technology (Indian School of Mines), Dhanbad, 826004 Jharkhand India

**Keywords:** Cardiovascular disease, ECG, Deep learning, Bidirectional LSTM, Explainable AI, Cardiology, Computational biology and bioinformatics, Diseases, Mathematics and computing

## Abstract

Cardiovascular disorders (heart diseases) are the most prevalent cause of death on a global scale. So early detection and classification increase the likelihood of survival. In the context of machine learning techniques, there is always a need for an accurate and explainable predictive model for detecting various diseases, such as cardiac disorders. The work carried out in this paper stacks bidirectional long short-term memory with deep learning to propose two models. The first model is used to detect cardiac disease with a binary label classification, while the second one classifies cardiac disease, which is a multi-label classification problem. Bidirectional LSTM is used as an approximate algorithm for feature extraction. Deep learning is used for classification purposes. The proposed models are trained and validated over the PTB-XL dataset. The performance of these models is evaluated and compared against state-of-the-art methods. The comparison shows the proposed model outperforms other methods in terms of accuracy, precision, f1-score, and recall. SHAP is used to make these models explainable, which in turn helps to annotate different diseases on the ECG report.

## Introduction

Globally, cardiovascular diseases (CVDs) are the most prevalent cause of mortality, claiming around 17.9 million lives annually^1^. Cardiovascular diseases encompass a wide range of conditions that affect the heart and blood vessels. More than 80% of CVD-caused deaths are due to strokes and heart attacks, with 30% of these early deaths occurring in people under 70 years of age. Unhealthy lifestyle habits, such as inactivity, poor diet, smoking, and excessive alcohol intake, are the main factors contributing to cardiovascular disease and stroke. Furthermore, without quick diagnosis and treatment, these conditions can worsen or become life-threatening. The first step in assessing and classifying patients with chest discomfort is an electrocardiogram (ECG). The ECG visually records the electrical activity of the heart. It is a quick, inexpensive, safe, painless and noninvasive procedure commonly used to diagnose conduction problems, arrhythmias, myocardial infarction, and ventricular hypertrophy. The ECG waves are labeled with the letters P, Q, R, S, T, and U. The P wave reflects atrial depolarization, while the QRS complex and the ST-T-U complex (which include the ST segment, the T wave, and the U wave) represent ventricular depolarization and repolarization, respectively^2^. The results of an ECG are immediately available and fairly affordable. Consequently, this method is vital for the diagnosis of heart problems and is of significant clinical importance. Although ECG machines are portable and suitable for both rural and urban settings, trained cardiologists are not always available in rural areas to interpret results and diagnose heart conditions. In addition, in emergencies, the diagnosis can lack accuracy; Even with rapid analysis, subtle changes in an ECG can be overlooked. The problem is amplified when noise interferes with ECG signals. This has led to research on machine learning (ML) models for the automatic diagnosis of heart disorders, since computational methods and ML algorithms can identify abnormalities in digital ECG data. Besides reducing misdiagnoses, implementing an effective ML model on ECG data can save a significant amount of time^3^. AI models often leave clinicians with a ‘‘black box” feeling because they lack transparency in decision-making processes^4,5^. The challenge of AI in clinical decision-making is that its results are not always straightforward to interpret. Since doctors are responsible for their decisions, AI models must be explainable if we aim to develop and implement cost-effective AI solutions in healthcare. Therefore, creating a reliable AI model capable of analyzing ECG data and providing visually interpretable results to assist cardiologists in diagnosing heart diseases is crucial. The main contributions of this paper are listed below.

1. Two approaches are proposed: one for detecting heart disease and the other for classification.
2. The models consist of stacking bidirectional long short-term memory (LSTM) and deep learning techniques. Bidirectional LSTM is used as an approximate algorithm for feature extraction, while deep learning is applied for classification.
3. The models are compared against different classifiers and state-of-the-art methods.
4. Additionally, the proposed model is designed as an explainable AI using SHAP, with healthcare professionals assisting in identifying various diseases in the ECG report.

## Related works

The study^6^ presents a systematic review of recent methods for cardiovascular disease detection and classification, highlighting advancements in machine learning and deep learning techniques. A spatio-temporal CNN with five layers was proposed in Ref. 2 for classifying different heart diseases. They trained and validated their proposed model on the PTB-XL and Arrhythmia Dataset, respectively. The work in Ref. 7 compares various convolutional neural network (CNN) models for heart disease classification, suggesting that inception- and ResNet-based architectures perform better in terms of accuracy. They used the ICBEB2018 and PTB-XL datasets. The work in Ref. 8 employed a CNN on the PTB-XL dataset for heart disease classification. They also demonstrated that using CNN with QRS complexes and entropy-based features provides higher accuracy than other methods. The work in Ref. 9 used a deep LSTM, trained and validated on the PTB-XL dataset, to detect one type of heart disease, namely myocardial infarction, with an accuracy of 77.12%. The author in Ref. 10 proposed a hybrid model combining Recurrent Unit (GRU) and Extreme Learning Machine (ELM), called CIGRU-ELM, to address the class imbalance problem across five classes of the PTB-XL dataset. They used this model to classify various heart diseases with an average accuracy of 0.89. A CNN with a non-local convolutional block attention module was proposed in Ref. 11 for heart disease classification. This model was trained separately on the MIT-BIH arrhythmia database and PTB-XL, achieving F1 scores of 0.9644 and 0.8507, respectively. In Ref. 12, features were extracted from the PTB-XL dataset using a CNN for heart disease classification; a random forest model then classified these features with an accuracy of 99.2%. The authors in Ref. 13 proposed a hybrid model consisting of two CNN layers and one SincNet network for heart disease classification, which was trained and validated on the PTB-XL dataset. A Multifeatured Transformer (MF-Transformer) model was introduced in Ref. 14 for classifying heart disease, achieving an accuracy of 75.82% by training and validating on the PTB-XL dataset. In Ref. 15, four classifiers–namely, Random Forest, Radial Basis Function, K-Nearest Neighbor, and Decision Tree–were applied to the PTB-XL dataset for binary heart disease classification. They concluded that Random Forest had the highest accuracy, which was further fine-tuned to 99.62%. A deep learning model based on loss optimization was proposed in Ref. 16 for multi-class cardiac disease classification, achieving an overall accuracy of 96%. In Ref. 17, Orthogonal Matching Pursuit (OMP) algorithms were used for cardiac disease classification, achieving accuracies of 90% for binary and 78% for multi-class classification, all trained and validated on the PTB-XL dataset. ResNet-50 was proposed in Ref. 18 for classifying multiple cardiac diseases, achieving an accuracy of 99% when trained and validated on PTB-XL. An explainable AI model for cardiac disease classification was detailed in Ref. 19, claiming an accuracy of 89%. The study in Ref. 20 extracted spectrograms from the PTB-XL dataset and fed them into CNNs for binary heart disease classification, achieving an accuracy of 99%. The widespread use of deep learning and its variants for classifying various cardiac diseases from ECG data is likely due to their ability to effectively extract key features during training. Among these variants, LSTM is especially suited for sequential input. Compared to LSTM, bidirectional LSTM(BiLSTM) has two networks: one processing past data in a forward direction and the other using future data in reverse, making it more effective at feature extraction. Additionally, as a recurrent neural network variant, bidirectional LSTM can approximate any algorithm^21^. The paper^22^ proposes a hybrid deep learning model combining M2MASC with CNN-BiLSTM to enhance the precision of heart disease classification. The study^23^ introduces a modified mixed attention mechanism integrated with a deep BiLSTM for enhanced multiclass heart disease classification. The authors propose^24^ a Deep Bidirectional long short-term memory classifier (SCN-Deep BiLSTM) model that captures both spatial and temporal features to improve heart disease prediction. This motivates us to propose a stacked model that combines bidirectional LSTM and deep learning techniques for classifying cardiac diseases from ECG data. Bidirectional long short-term memory is used as an approximate algorithm for feature extraction, while deep learning is employed for classification purposes.

## Dataset

The PTB-XL dataset^21,25^ consists of 21837 clinical 12-lead ECG records with a duration of 10 s from 18,885 patients. The patient population is split into 52 percent males and 48 percent women, with ages ranging from 0 to 95 years (median 62 and interquartile range 22).

**Fig. 1:**
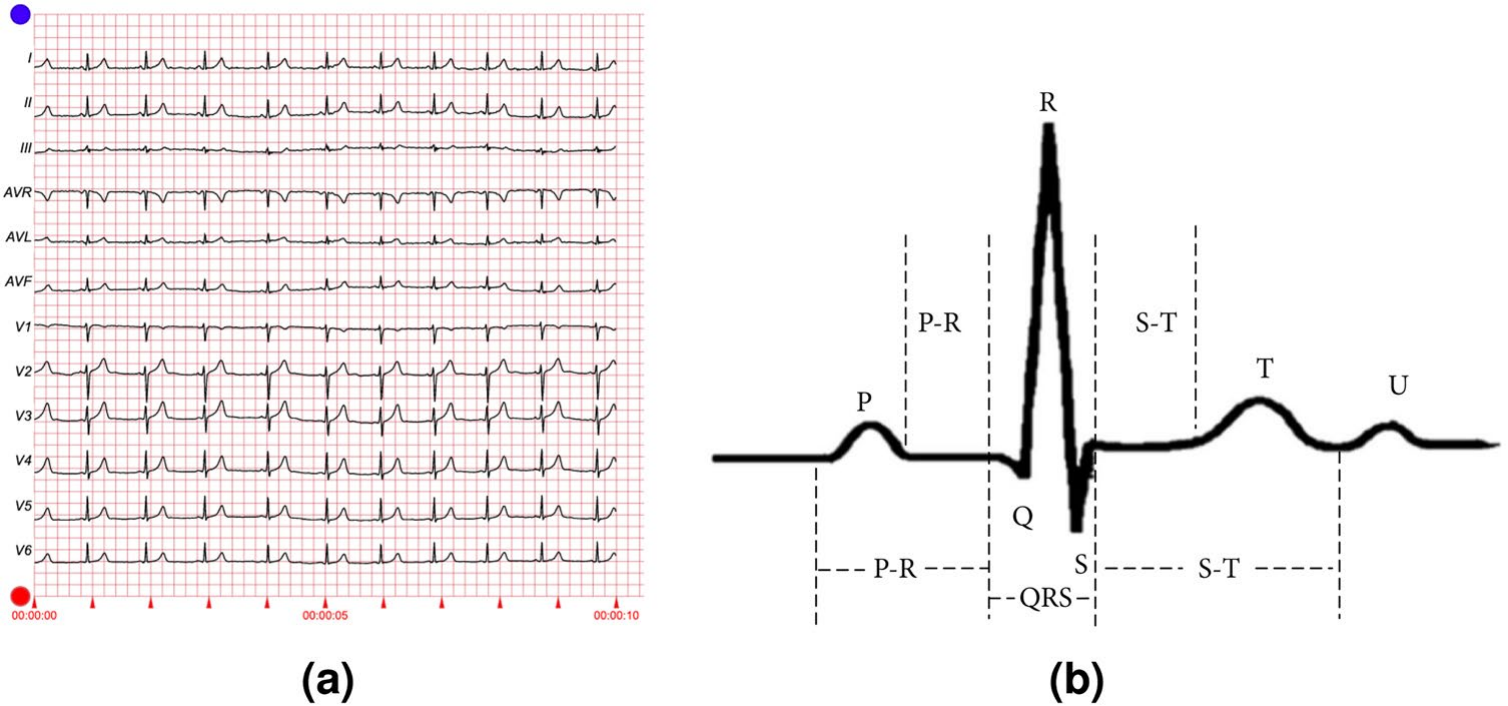
A sample ECG report.

A sample ECG report is shown in Fig. 1, consisting of an ideal waveform in Fig. 1a and a single heartbeat waveform in Fig. 1b . The training and validation data (Table 1) are prepared from the dataset mentioned above separately for binary classification and multi-class classification as follows:

1. For binary classification purposes, the number of records containing the normal ECG and not normal ECG is the same, i.e. 9517. The number of records for training and validation is 6662 and 2855, respectively.
2. The training and validation datasets are prepared class-wise, with a split ratio of 70% and 30%, respectively, totaling the number of records for training and validation (Table 1).

**Table 1:** Different heart disease types along with their instances.

Type	Class	Numeric value	No. of records	Number of records	
				Training	Validation
Normal ECG	NORM	0	9517	6662	2855
Myocardial infarction	MI	1	5473	3832	1641
ST/T Change	STTC	2	5237	3666	1571
Conduction disturbance	CD	3	4901	3431	1470
Hypertrophy	HYP	4	2649	1855	794
				19446	8331

### Patient-wise split of training, validation, and testing data

Unlike spit on the instance side of the data set, the data set is now split into patient-wise to train, validate, and test the data set. The details are presented in Table 2.

**Table 2:** Different heart disease types along with their instances.

Type	Class	Numeric value	No. of records	Number of records		
				Training	Validation	Testing
Normal ECG	NORM	0	9528	6627	1467	1434
Myocardial infarction	MI	1	5441	3809	794	838
ST/T Change	STTC	2	2823	1985	430	408
Conduction disturbance	CD	3	2327	1662	315	350
Hypertrophy	HYP	4	1311	906	202	203
				14989	3208	3233

## Data preprocessing

To ensure the electrocardiogram (ECG) signals are suitable for deep learning, several preprocessing steps are applied: sampling, filtering, normalization, windowing, padding, and trimming. Each step has a mathematical foundation, described below.

### Sampling frequency

The continuous-time ECG signal $x_c(t)$ is sampled at a frequency $f_s$ to obtain a discrete-time signal *x*[*n*]:

1 $$\begin{aligned} x[n] = x_c(nT_s), \quad T_s = \frac{1}{f_s}, \end{aligned}$$

where $T_s$ is the sampling period. According to the Nyquist theorem,

2 $$\begin{aligned} f_s \ge 2 f_{\max } \end{aligned}$$

with $f_{\max }$ being the maximum frequency component of the ECG (typically 150 Hz)^26^.

### Filtering

To remove baseline wander and high-frequency noise, a bandpass filter (e.g. 0.5–40 Hz) is applied:

3 $$\begin{aligned} y[n] = \sum _{k=0}^{M} b_k , x[n-k] - \sum _{j=1}^{N} a_j , y[n-j], \end{aligned}$$

where $b_k$ and $a_j$ are the filter coefficients^27^.

### Normalization

Normalization ensures consistent amplitude scaling across signals. Two common approaches are 

*Min–Max normalization*

4 $$\begin{aligned} x_{\text {norm}}[n] = \frac{x[n] - \min (x)}{\max (x) - \min (x)}. \end{aligned}$$

*Z-score normalization*

5 $$\begin{aligned} x_{\text {norm}}[n] = \frac{x[n] - \mu }{\sigma }, \end{aligned}$$

where $\mu$ is the mean and $\sigma$ is the standard deviation^28^.

### Windowing

ECG signals are segmented into fixed-length windows using a window function *w*[*n*]:

6 $$\begin{aligned} x_w[n] = x[n] \cdot w[n], \quad 0 \le n \le L-1. \end{aligned}$$

For instance, the Hamming window is defined as

7 $$\begin{aligned} w[n] = 0.54 - 0.46 \cos \left( \frac{2\pi n}{L-1}\right) , \end{aligned}$$

which reduces spectral leakage^29^.

### Padding

To standardize sequence lengths, zero-padding is applied:

8 $$\begin{aligned} x_p[n] = {\left\{ \begin{array}{ll} x[n], & 0 \le n< L, \\ 0, & L \le n < L_p \end{array}\right. } \end{aligned}$$

where *L* is the original length and $L_p$ is the padded length^30^.

### Trimming

If the ECG sequence is longer than required, it is truncated:

9 $$\begin{aligned} x_t[n] = x[n], \quad 0 \le n < L_t, \end{aligned}$$

where $L_t$ is the target trimmed length^31^.

## BLSTM-DNN- A stacked model of Bidirectional LSTM and DNN for heart disease detection and its classification

**Fig. 2:**
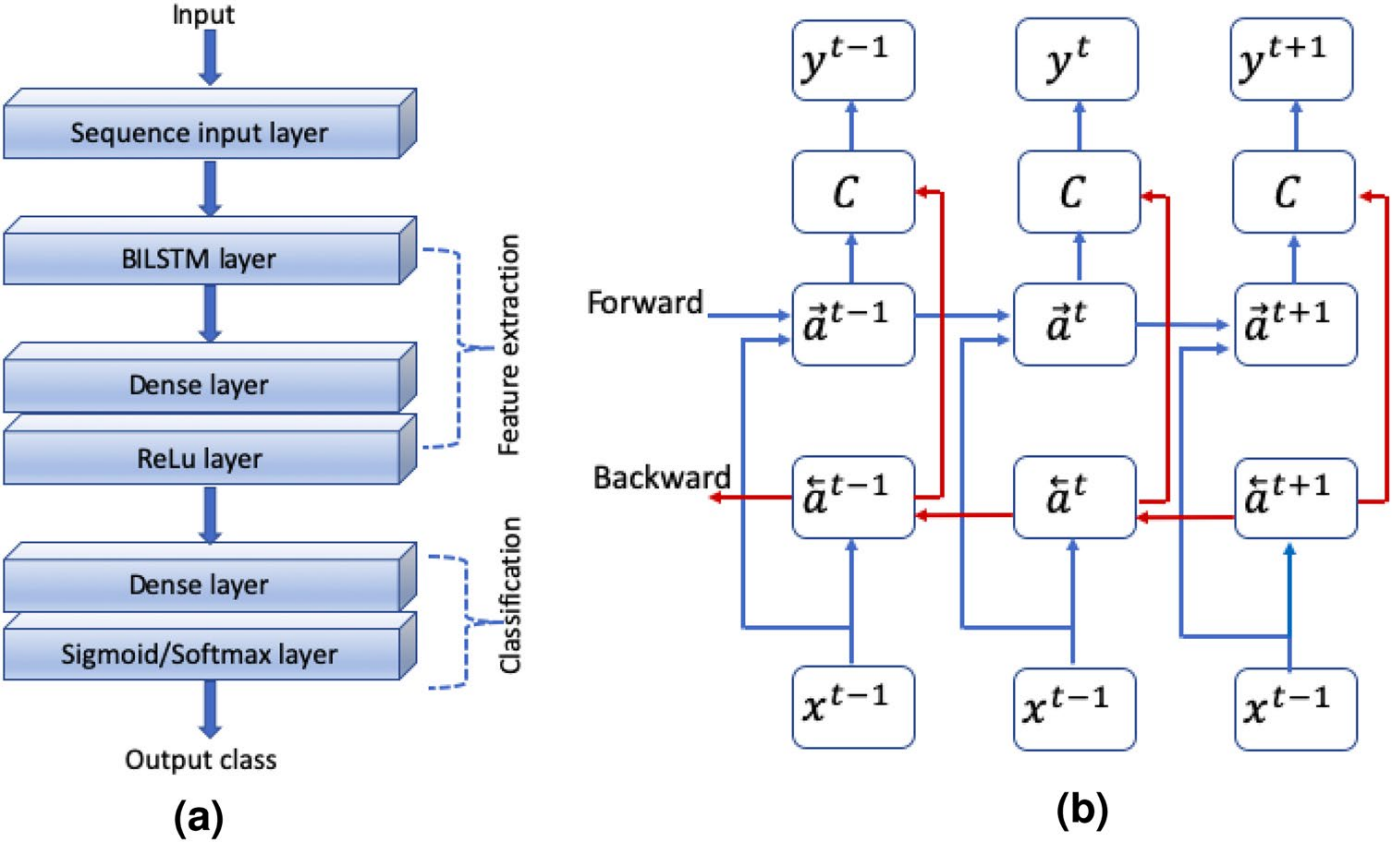
BLSTM-DNN: A combined model of Bidirectional LSTM and DNN for heart disease detection and its classification.

The proposed model is a stacked model of bidirectional LSTM and deep neural network (Fig. 2a). The proposed model consists of a sequence input layer as the first layer, followed by a bidirectional LSTM layer and two dense layers. The feature extraction is performed by stacking the output of the bidirectional LSTM layer into the first dense layer. The second dense layer is meant for classification purposes. The sigmoid activation layer is used for binary classification, whereas the softmax layer is used for multi-class classification. The intuitive reason to combine these two techniques is explained below.

### Bidirectional LSTM as an approximation for a feature extraction algorithm

Bidirectional LSTM is a recurrent neural network that allows input data to flow in both the forward and backward directions. Thus, the input sequence holds information related to its present and past.

If *f* is a computable function, there exists a finite recurrent neural network (RNN) that can compute *f*. Furthermore, certain finite RNNs are Turing complete, and hence, can approximate any algorithm^32^. This, in turn, implies that bidirectional long short-term memory (BiLSTM) networks can also approximate any algorithm. Therefore, BiLSTMs can be effectively implemented for feature extraction from ECG data. The working principle of a bidirectional LSTM is illustrated in Fig. 2b for better insight.

At each time *t*, the LSTM takes the following three inputs: $x^{t}$ from the input layer, $a^{t-1}$ the output of the previous layer, and $c^{t-1}$ the output of the previous cell. The outputs produced are $a^{t}$ and $c^{t}$, representing the new activation and new cell state, respectively. Each LSTM cell contains three gates: an update gate $\digamma _{u}$, a forget gate $\digamma _{f}$, and an output gate $\digamma _{o}$. The values of these gates at time *t* can be updated by:

10 $$\begin{aligned} \digamma _u= & \sigma (\omega _u[a^{<t-1>}, x^{<t>}]+ \beta _u), \end{aligned}$$

11 $$\begin{aligned} \digamma _f= & \sigma (\omega _f[a^{<t-1>}, x^{<t>}]+ \beta _f), \end{aligned}$$

12 $$\begin{aligned} \digamma _o= & \sigma (\omega _o[a^{<t-1>}, x^{<t>}]+ \beta _o), \end{aligned}$$

where $\omega _k$, $k\epsilon \{u, f, o\}$ are the weight matrices associated with the update gate, forget gate, and output gate, respectively. Similarly, $\beta _k$, $k\epsilon \{u, f, o\}$ is the bias. $\sigma ()$ and *tanh*() are the sigmoid and hyperbolic tangent activation functions. The new candidate value $c^{N<t>}$ to replace the cell can be written as

13 $$\begin{aligned} c^{N<t>}= tanh (\omega _c[a^{<t-1>}, x^{<t>}]+ \beta _c). \end{aligned}$$

The two outputs obtained from the LSTM can be expressed as

14 $$\begin{aligned} c^{<t>}= & \digamma _u \times c^{N<t>} + \digamma _f \times c^{<t-1>}, \end{aligned}$$

15 $$\begin{aligned} a^{<t>}= & \digamma _o \times c^{<t>}. \end{aligned}$$

### Deep neural network (DNN)

Deep learning is an artificial neural network consisting of multiple computational layers that learns by exploring the intricate structure of input data. Due to its architecture, it inherently reduces data complexity through implicit feature extraction and dimensional reduction. Furthermore, it is more suitable to handle non-linear type input data.

### Proposed model for heart disease detection (BLSTM-DNN-BC)

**Fig. 3:**
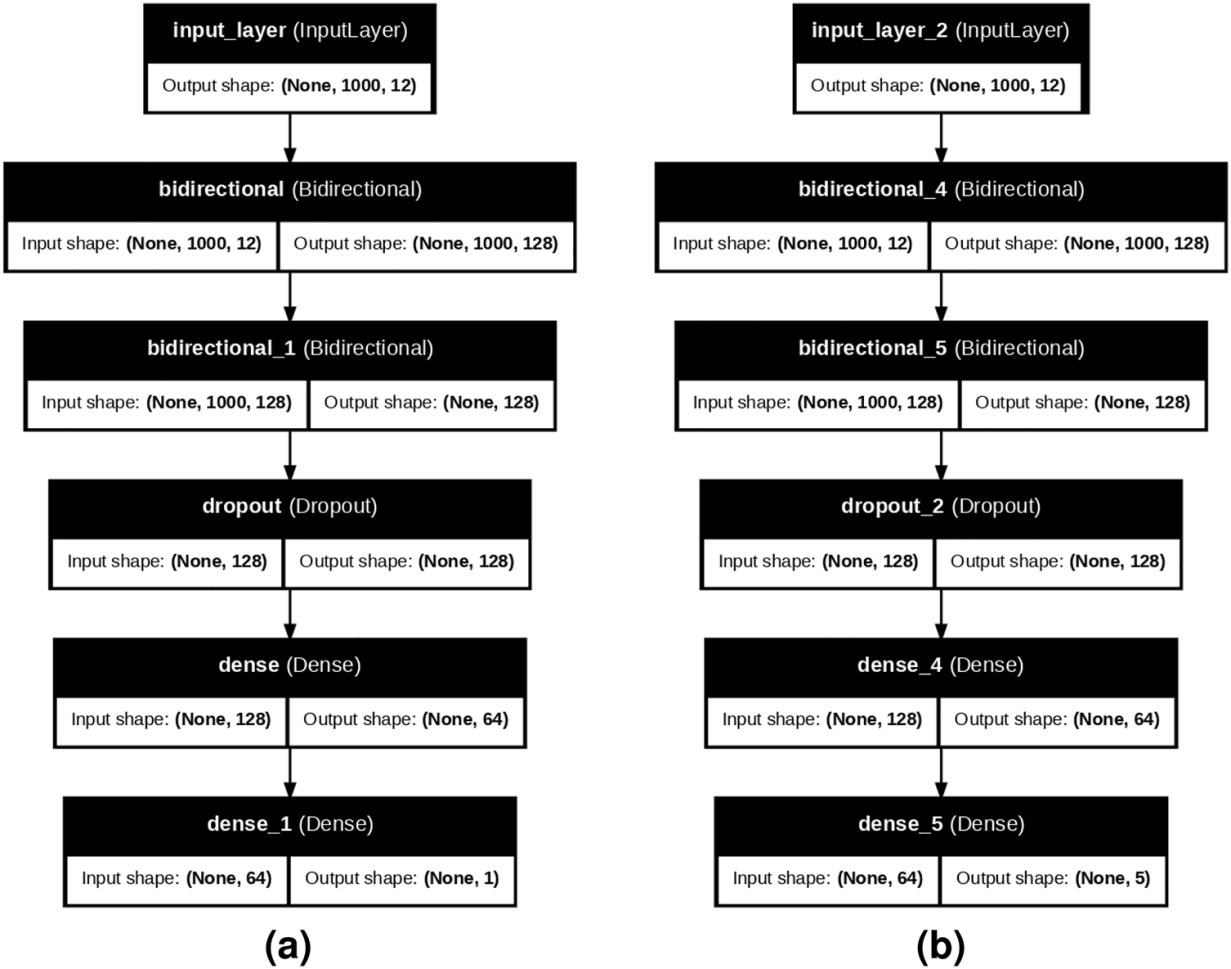
BLSTM-DNN A combined model of Bidirectional LSTM and DNN for heart disease detection.

The proposed model for heart disease detection (Fig. 3a) consists of one input layer, one bidirectional LSTM layer, and two dense layers. The bidirectional layer processes the input (None, 1000, 12) to provide the output (None, 64). Similarly, the dense layer further reduces the features to (None, 8), which is eventually used by the second dense layer to detect heart disease. The total number of trainable parameters of the proposed model is 153,920.

### Proposed model for heart disease classification (BLSTM-DNN-MC)

The proposed model for the classification of heart disease (Fig. 3b) has an architecture similar to that explained above for binary classification. However, the first dense layer provides an output (none, 8) which is further processed by the second dense layer to classify the five different heart diseases listed in Table 1.

## Simulated results and discussion

**Fig. 4:**
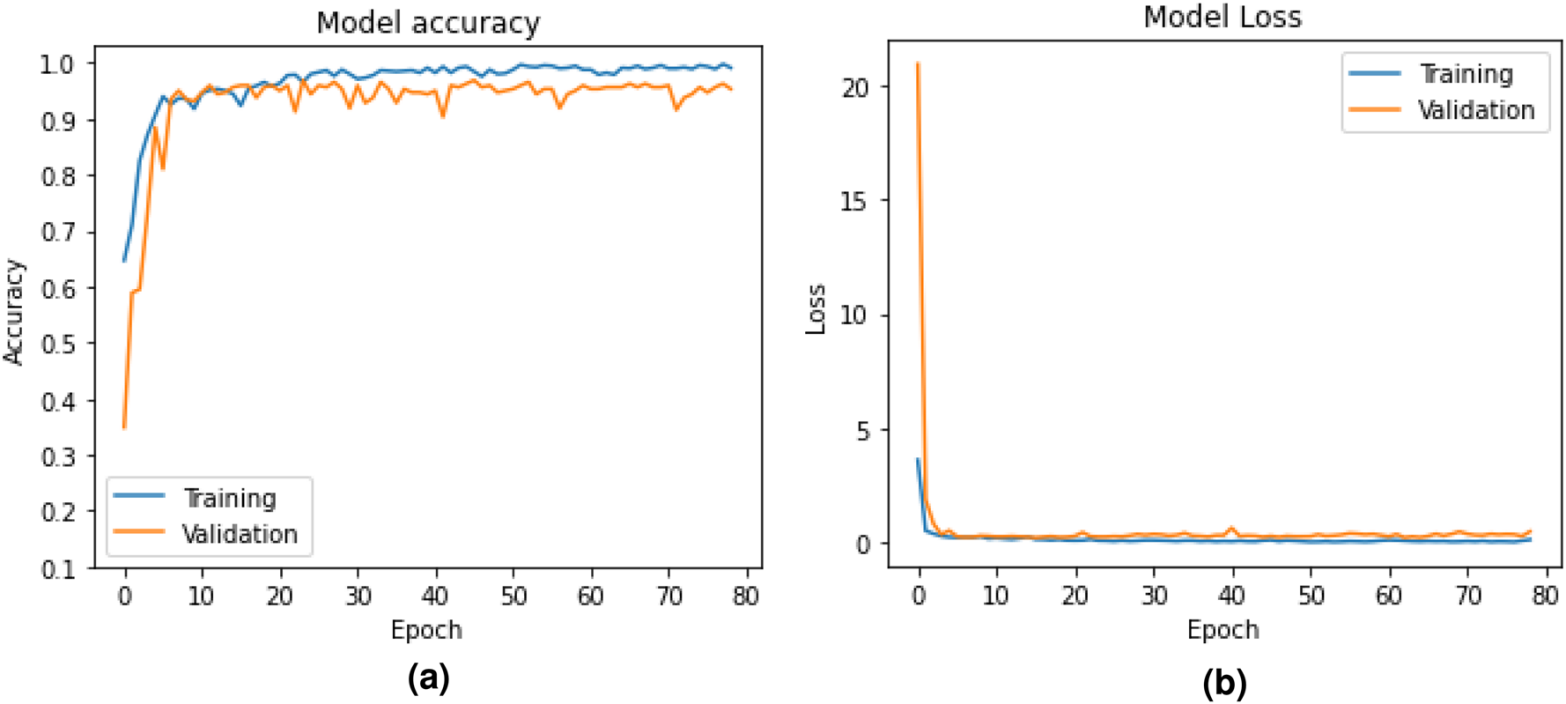
Accuracy and loss of BLSTM-DNN-BC during training and validation.

**Fig. 5:**
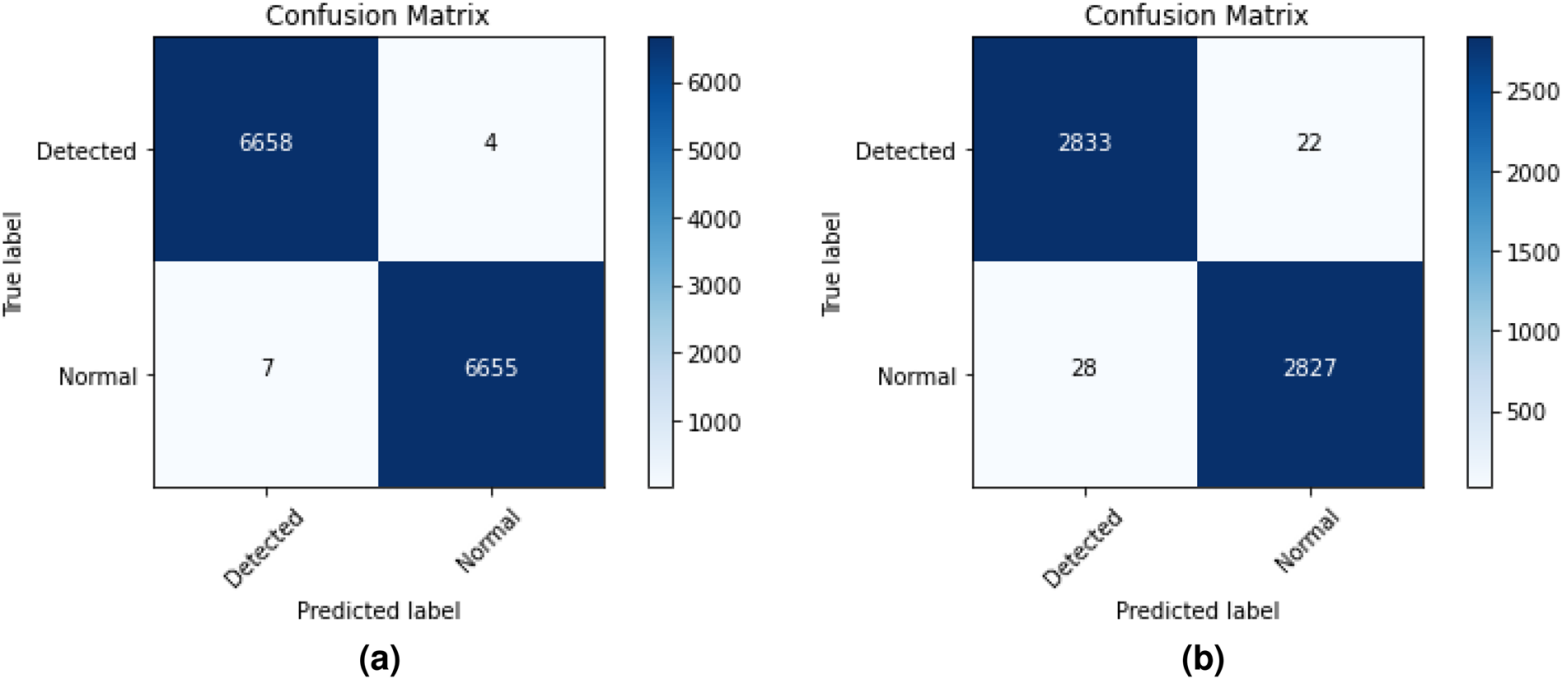
Confusion matrices for training and validation of BLSTM-DNN-BC.

The simulation of the proposed models is carried out in a Google Colab environment with GPU support as needed. The accuracy and loss curves generated during the training and validation of BLSTM-DNN-BC are shown in Fig. 4. Similarly, for model BLSTM-DNN-MC, these are shown in Fig. 5. These figures, on the one hand, ensure that neither of the models suffers from data overfitting, while on the other hand, they ensure that both models predict with high accuracy. The performance of both models is evaluated using the following performance metrics.

1. Precision (P) - $P=\frac{TP}{FP}$, where *TP* is the true positive and *FP* is the false positive
2. Recall (R) - $R=\frac{TP}{FN}$, where *FN* is the false negative
3. F1-score - $$F1-score=\frac{2\times P \times R }{P+R}$$
4. Receiver operating characteristic curve (ROC)

Implementation details of the proposed models The network parameters of the proposed models are presented in Table 3.

**Table 3:** Network parameters of the proposed models.

Parameter	BLSTM-DNN-BC	LSTM-DNN-MC
Number of BLSTM units	2 layers, 128 units each	2 layers, 128 units each
Hidden layers	1 layer, 64 units	1 layer, 64 units
Dropout	0.5	0.5
Activation functions	ReLU, Sigmoid	ReLU, Softmax
Optimizer	Adam	Adam
Learning rate	0.005	0.05
Batch size	256	256
Epochs	80	80
Early stopping	Yes	Yes
Parameter counts	439,685 (1.68 MB)	440,465 (1.68 MB)
Trainable params	146,561 (572.5 KB)	146,821 (573.5 KB)
Non-trainable params	0 (0.00 B)	0 (0.00 B)
Optimizer params	293,124 (1.12 MB)	293,644 (1.12 MB)
Total Parameters + Memory	439,685 (1.68 MB)	440,465 (1.68 MB)

### Performance of BLSTM-DNN-BC

The different performance measurement of the proposed BLSTM-DNN-BC model for heart disease detection is presented in Table 4. The confusion matrices for training and validation are shown in Fig. 5.

The precision, recall, F1 score, and accuracy generated by the proposed BLSTM-DNN-BC model are presented in Table 4 for both training and validation on the respective datasets. This process is repeated five times, and the mean and standard deviation are computed.

**Table 4:** Performance of proposed BLSTM-DNN-BC model during training and validation.

	*P*	R	F1-score	Accuracy
Training	1.00–0.01	0.99 ± 0.001	1.00–0.01	0.995 ± 0.005
Validation	0.99 ± 0.001	0.99 ± 0.001	0.99 ± 0.001	0.99 ± 0.001

**Fig. 6:**
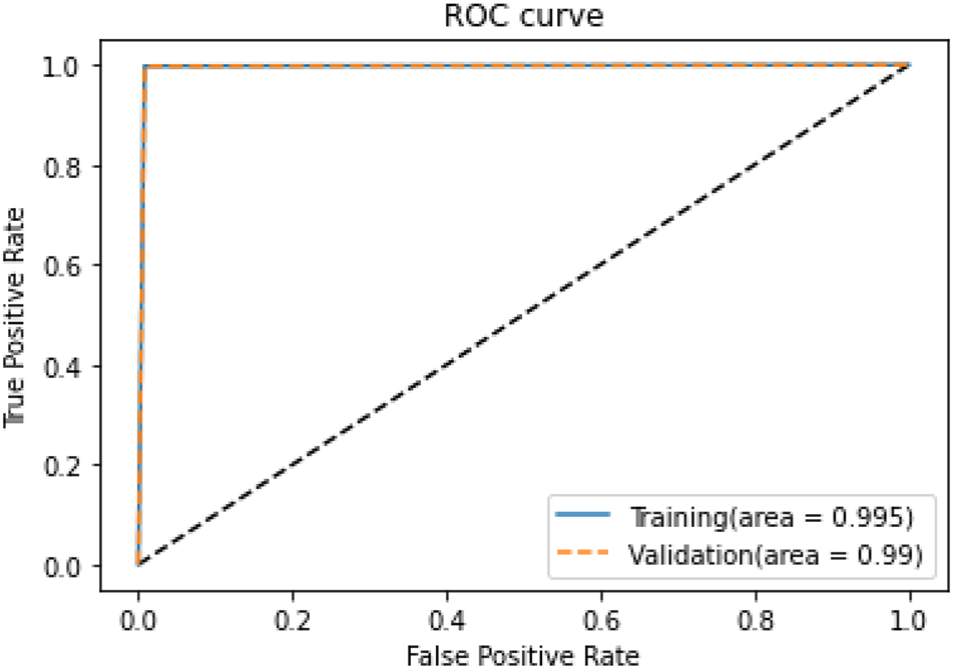
ROC of the proposed BLSTM-DNN-BC during training and validation.

The ROC of the proposed BLSTM-DNN-BC during training and validation is depicted in Fig. 6.

The performance of the proposed BLSTM-DNN-BC achieves an accuracy of 99%, as evident from the performance metrics presented in this section. Thus, it is quite robust for heart disease detection if employed for clinical trials.

#### Performance of BLSTM-DNN-MC

**Fig. 7:**
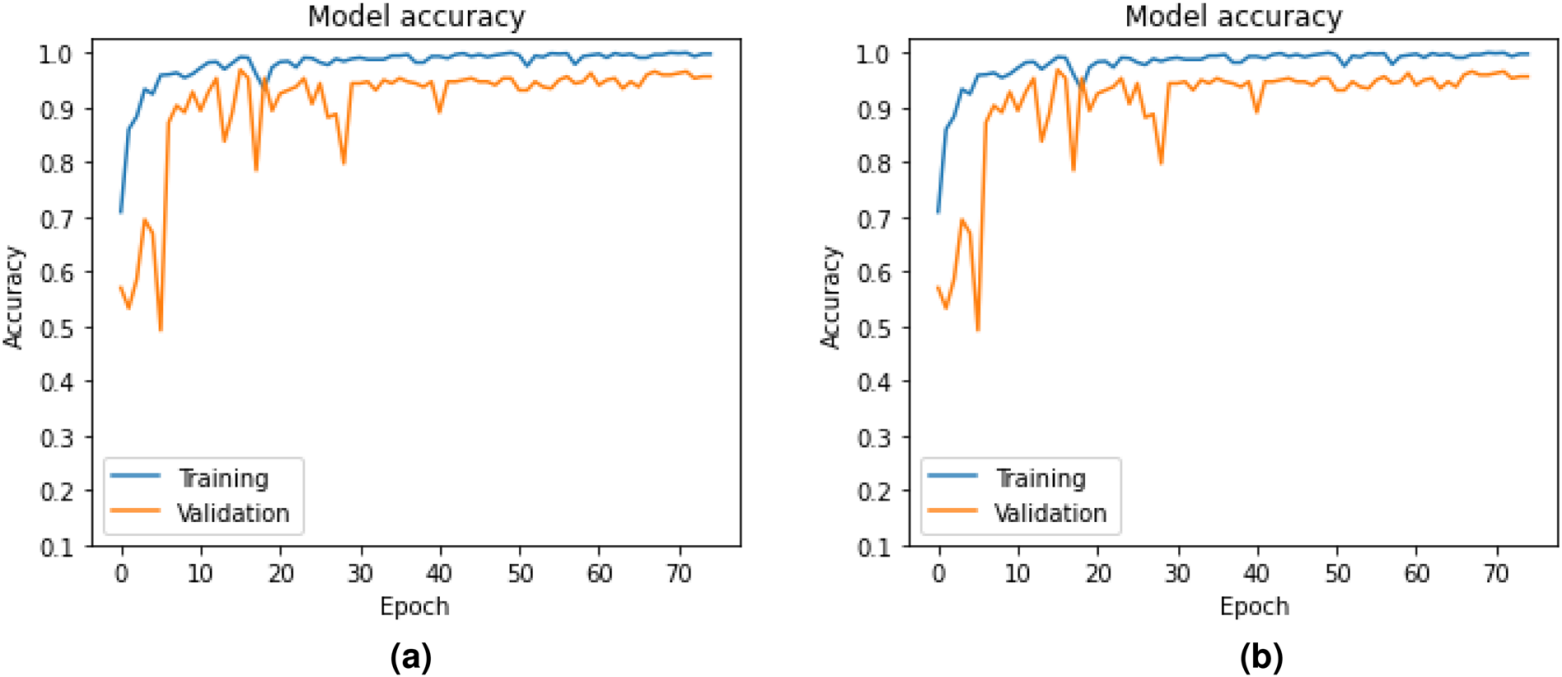
Accuracy and loss of BLSTM-DNN-MC during training and validation.

Heart disease classification is a multi-class problem with five different major classes. The proposed BLSTM-DNN-MC model is trained and validated for this classification purpose by using the data mentioned in Table 5. The respective accuracy and loss obtained during training and validation are plotted in Fig. 7a and b.

**Table 5:** Performance of proposed BLSTM-DNN-MC model during training and validation.

	Class	*P*	R	F1-score	Macro-F1	Accuracy
Training	0	0.99 ± 0.001	0.98 ± 0.001	0.99 ± 0.001	0.992 ± 0.005	0.995 ± 0.005
	1	1.00-0.001	0.99 ± 0.001	1.00-0.01		
	2	1.00-0.001	0.99 ± 0.001	1.00 -0.01		
	3	1.00-0.001	0.95 ± 0.001	0.99 ± 0.001		
	4	1.00-0.01	0.95 ± 0.001	0.98 ± 0.001		
Validation	0	0.98 ± 0.001	0.97 ± 0.001	0.98 ± 0.001	0.98 ± 0.005	0.99 ± 0.005
	1	1.00-0.001	0.99 ± 0.001	1.00-0.01		
	2	1.00-0.001	0.99 ± 0.001	1.00-0.01		
	3	0.93 ± 0.001	0.99 ± 0.001	0.96 ± 0.001		
	4	1.00-0.001	0.93±0.001	0.96 ± 0.001		

**Fig. 8:**
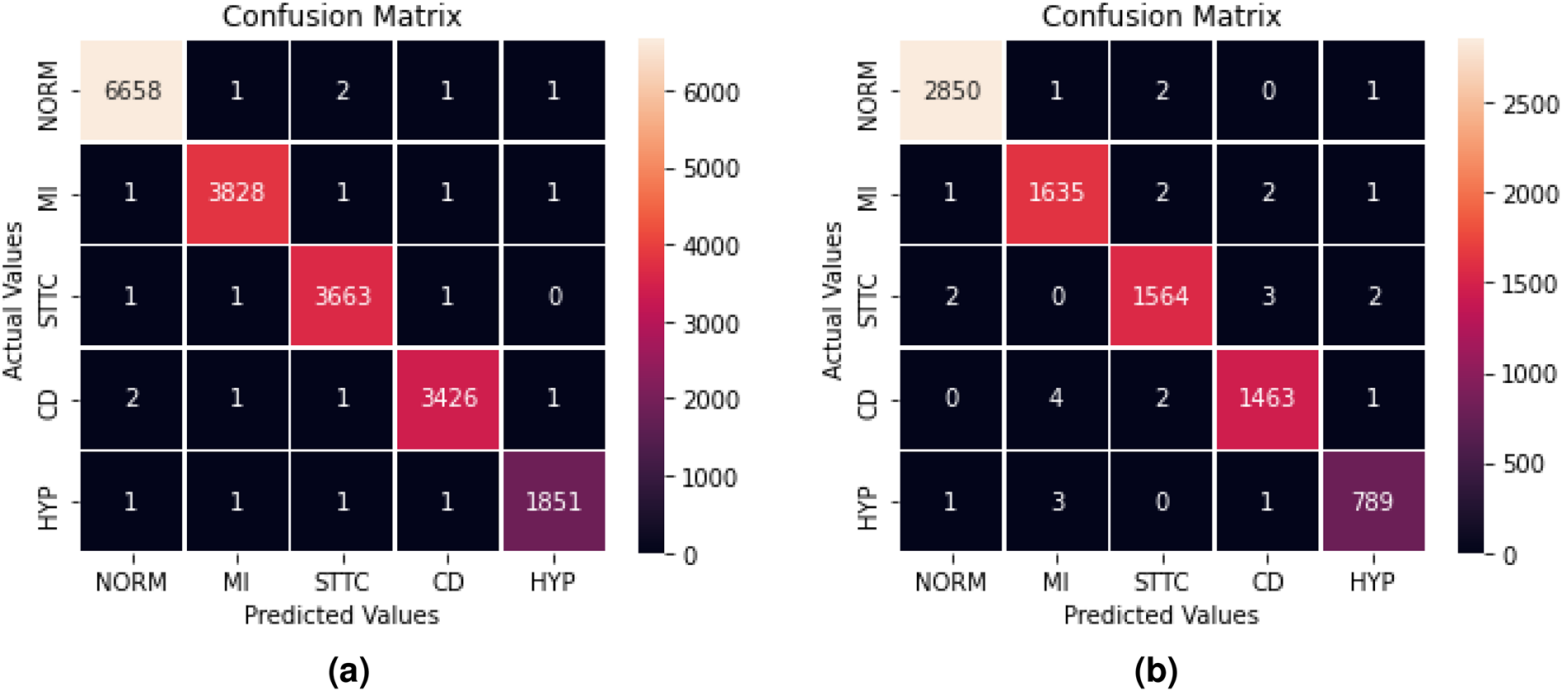
Confusion matrices for training and validation of BLSTM-DNN-MC.

**Fig. 9:**
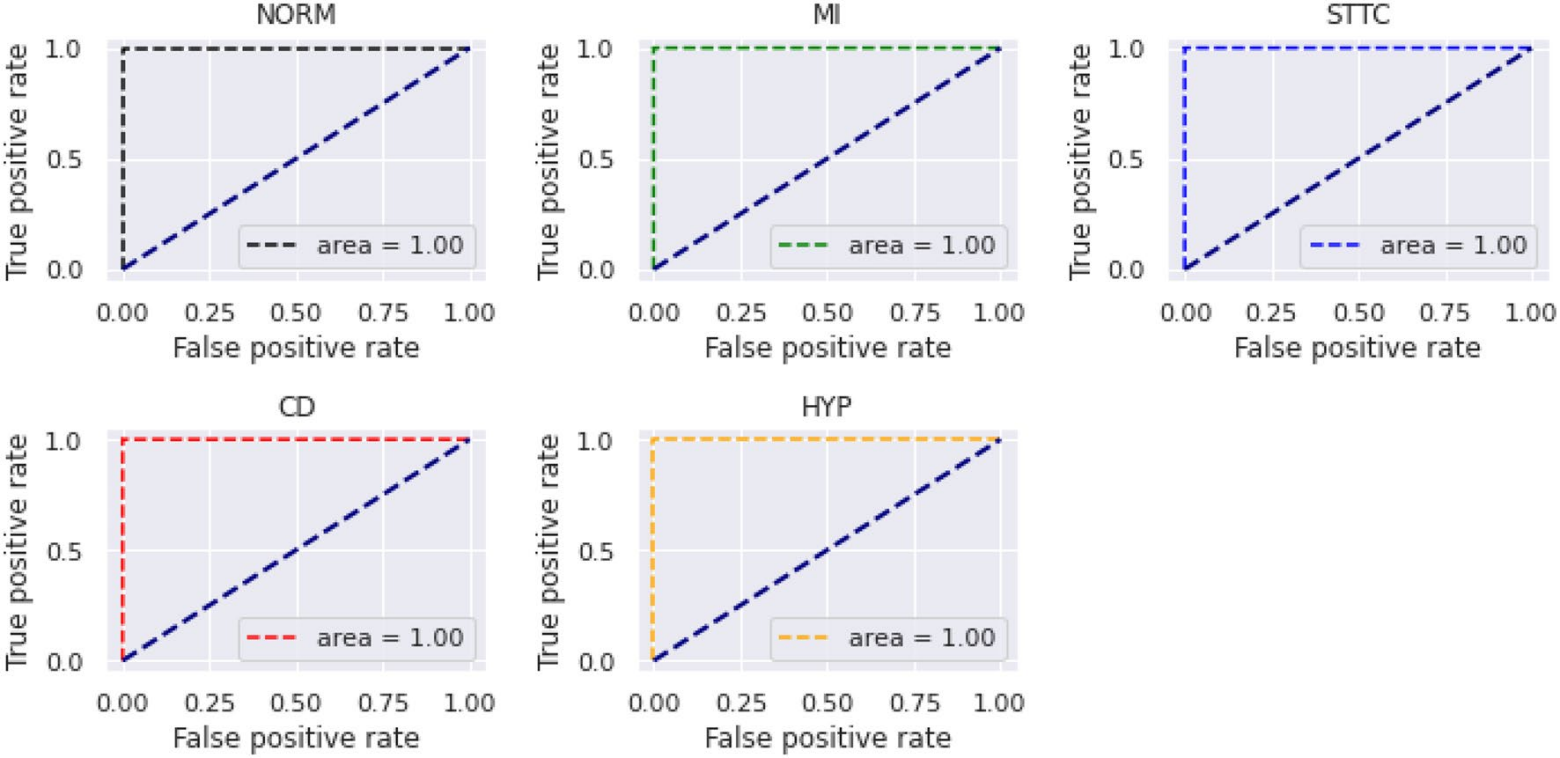
ROC of the proposed BLSTM-DNN-MC during training and validation.

The confusion matrix generated during training and validation of the proposed model is shown in Fig. 8. The ROC curve for the proposed BLSTM-DNN-MC is shown in Fig. 9.

### Comparison with state-of-the-art methods

**Fig. 10:**
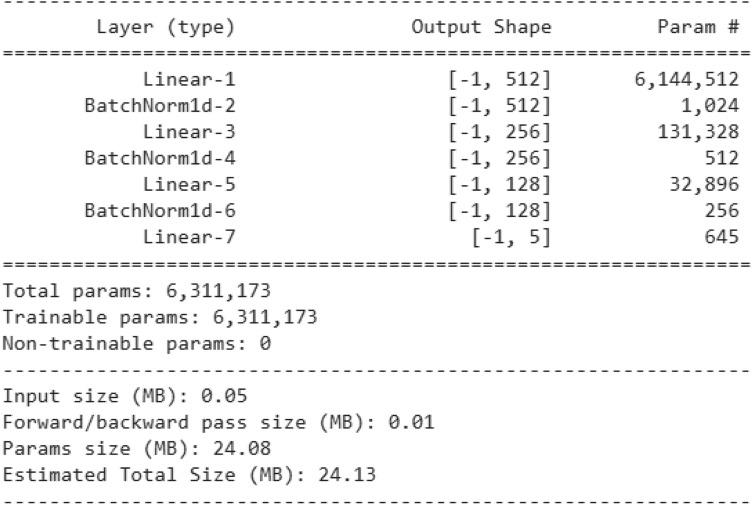
Architecture of DNN model.

**Fig. 11:**
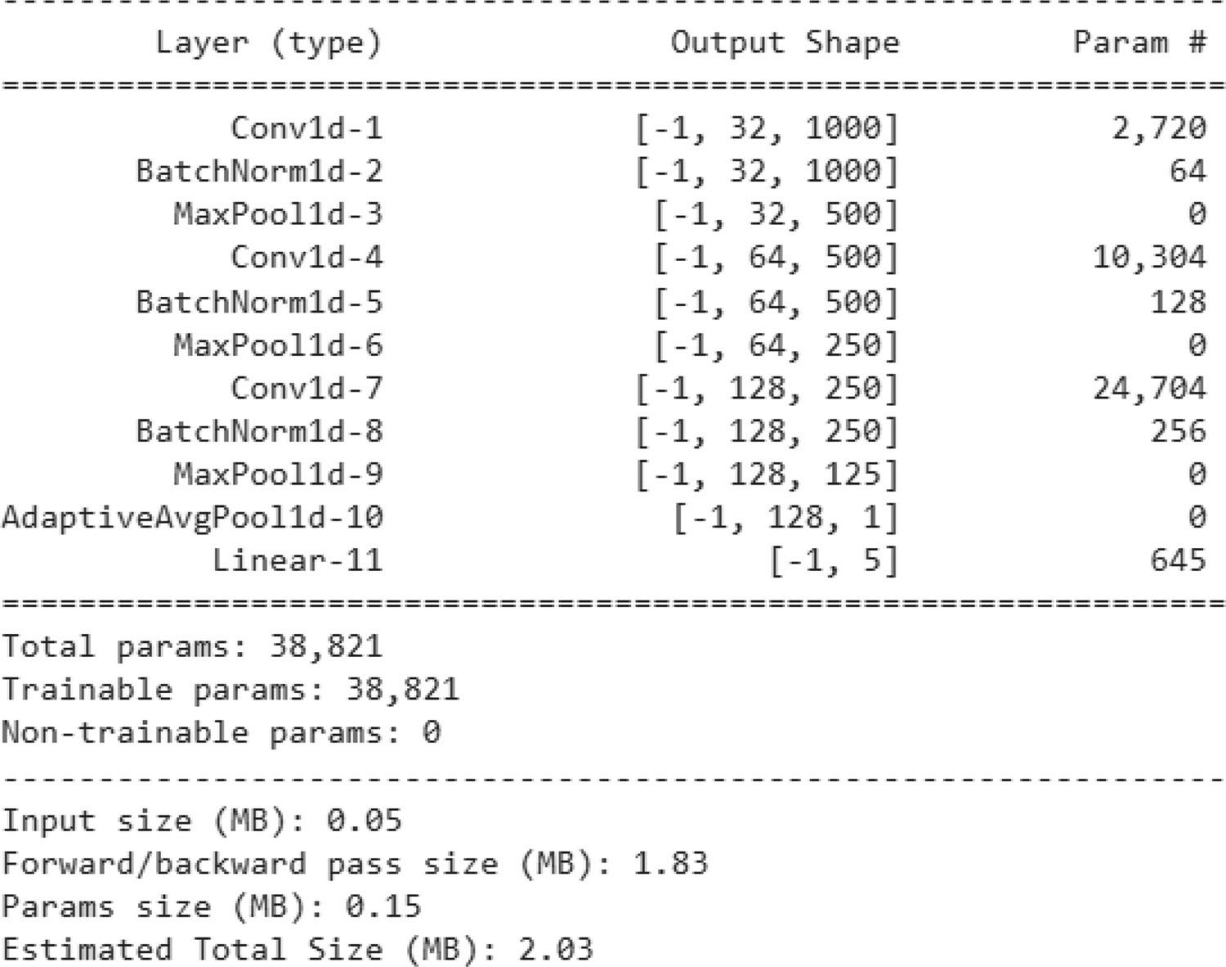
Architecture of CNN model.

**Fig. 12:**
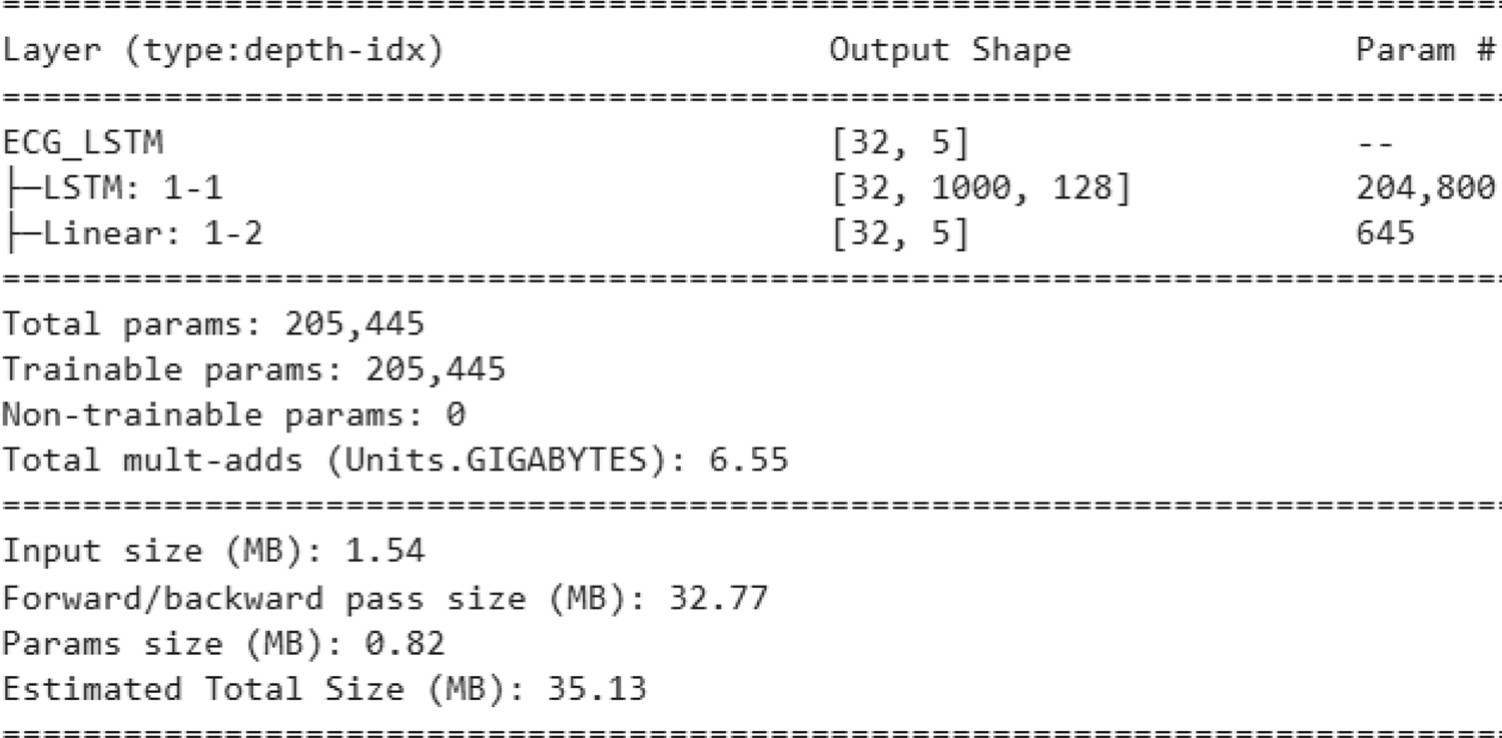
Architecture of LSTM model.

The proposed methods are compared with some standard stand-alone classifiers such as Deep neural network (DNN) (Fig. 10), Convolutional neural network (CNN) (Fig. 11), and LSTM (Fig. 12), as well as some hybrid classifiers like Deep neural network and support vector machine (DNN-SVM), neural network and random forest (DNN-RF), convolutional neural network and support vector machine (CNN-SVM). The results of these comparisons are presented in Tables 6 and 7, respectively.

**Table 6:** Comparison of proposed BLSTM-DNN-BC against state-of-the-art classifiers.

Model	Precision	Recall	F1-score	Accuracy	AUROC	AUPRC	MCC/Kappa
DNN	0.90 ± 0.001	0.87 ± 0.001	0.88 ± 0.001	0.89 ± 0.001	0.89 ± 0.005	0.88 ± 0.005	0.87 ± 0.005
DNN-SVM	0.92 ± 0.001	0.90 ± 0.001	0.90 ± 0.001	0.91 ± 0.001	0.91 ± 0.005	0.90 ± 0.005	0.89 ± 0.005
DNN-RF	0.92 ± 0.001	0.91 ± 0.001	0.91 ± 0.001	0.91 ± 0.001	0.92 ± 0.005	0.91 ± 0.005	0.90 ± 0.005
LSTM	0.89 ± 0.001	0.85 ± 0.001	0.88 ± 0.001	0.87 ± 0.001	0.88 ± 0.005	0.87 ± 0.005	0.86 ± 0.005
CNN	0.93 ± 0.001	0.91 ± 0.001	0.92 ± 0.001	0.93 ± 0.001	0.93 ± 0.005	0.92 ± 0.005	0.91 ± 0.005
CNN-SVM	0.97 ± 0.001	0.95 ± 0.001	0.96 ± 0.001	0.96 ± 0.001	0.96 ± 0.005	0.95 ± 0.005	0.94 ± 0.005
BLSTM-DNN-BC	0.99 ± 0.001	0.99 ± 0.001	0.99 ± 0.001	0.99 ± 0.001	0.99 ± 0.005	0.99 ± 0.005	0.99 ± 0.005

**Table 7:** Comparison of proposed BLSTM-DNN-MC against state-of-the-art classifiers.

Model	Class	P	R	F1-score	Macro-F1	Accuracy	Macro-AUROC	AUPRC	MCC	Kappa
DNN	0	0.895 ± 0.005	0.875 ± 0.005	0.885 ± 0.005	–	–	0.885	0.875	0.715	0.705
	1	0.905 ± 0.005	0.895 ± 0.005	0.905 ± 0.005	–	–	0.895	0.885	0.725	0.715
	2	0.915 ± 0.005	0.895 ± 0.005	0.905 ± 0.005	0.893 ± 0.005	0.895 ± 0.005	0.905	0.895	0.735	0.725
	3	0.905 ± 0.005	0.885 ± 0.005	0.895 ± 0.005	–	–	0.895	0.875	0.725	0.715
	4	0.885 ± 0.005	0.875 ± 0.005	0.875 ± 0.005	–	–	0.875	0.865	0.705	0.695
DNN-SVM	0	0.895 ± 0.005	0.915 ± 0.005	0.915 ± 0.005	–	–	0.915	0.905	0.745	0.735
	1	0.905 ± 0.005	0.915 ± 0.005	0.905 ± 0.005	–	–	0.915	0.915	0.755	0.745
	2	0.905 ± 0.005	0.905 ± 0.005	0.905 ± 0.005	0.907 ± 0.005	0.905 ± 0.005	0.925	0.915	0.765	0.755
	3	0.885 ± 0.005	0.905 ± 0.005	0.895 ± 0.005	–	–	0.905	0.905	0.735	0.725
	4	0.895 ± 0.005	0.925 ± 0.005	0.915 ± 0.005	–	–	0.925	0.915	0.755	0.745
DNN-RF	0	0.905 ± 0.005	0.935 ± 0.005	0.915 ± 0.005	–	–	0.935	0.925	0.795	0.785
	1	0.945 ± 0.005	0.955 ± 0.005	0.935 ± 0.005	–	–	0.955	0.945	0.815	0.805
	2	0.955 ± 0.005	0.955 ± 0.005	0.955 ± 0.005	0.947 ± 0.005	0.915 ± 0.005	0.965	0.955	0.835	0.825
	3	0.935 ± 0.005	0.955 ± 0.005	0.945 ± 0.005	–	–	0.955	0.945	0.815	0.805
	4	0.985 ± 0.005	0.985 ± 0.005	0.985 ± 0.005	–	–	0.985	0.975	0.875	0.865
LSTM	0	0.925 ± 0.005	0.945 ± 0.005	0.935 ± 0.005	–	–	0.945	0.935	0.815	0.805
	1	0.965 ± 0.005	0.915 ± 0.005	0.955 ± 0.005	–	–	0.955	0.945	0.825	0.815
	2	0.985 ± 0.005	0.975 ± 0.005	0.975 ± 0.005	0.957 ± 0.005	0.945 ± 0.005	0.975	0.965	0.845	0.835
	3	0.965 ± 0.005	0.975 ± 0.005	0.975 ± 0.005	–	–	0.975	0.965	0.845	0.835
	4	0.995	0.955 ± 0.005	0.945 ± 0.005	–	–	0.985	0.975	0.855	0.845
CNN	0	0.895 ± 0.005	0.905 ± 0.005	0.895 ± 0.005	–	–	0.905	0.895	0.735	0.725
	1	0.945 ± 0.005	0.915 ± 0.005	0.925 ± 0.005	–	–	0.945	0.935	0.805	0.795
	2	0.945 ± 0.005	0.925 ± 0.005	0.945 ± 0.005	0.925 ± 0.005	0.925 ± 0.005	0.955	0.945	0.815	0.805
	3	0.965 ± 0.005	0.955 ± 0.005	0.945 ± 0.005	–	–	0.965	0.955	0.835	0.825
	4	0.995	0.955 ± 0.005	0.915 ± 0.005	–	–	0.965	0.945	0.825	0.815
CNN-SVM	0	0.965 ± 0.005	0.945 ± 0.005	0.945 ± 0.005	–	–	0.965	0.955	0.845	0.835
	1	0.985 ± 0.005	0.975 ± 0.005	0.965 ± 0.005	–	–	0.975	0.965	0.865	0.855
	2	0.975 ± 0.005	0.935 ± 0.005	0.955 ± 0.005	0.953 ± 0.005	0.955 ± 0.005	0.975	0.955	0.855	0.845
	3	0.955 ± 0.005	0.945 ± 0.005	0.945 ± 0.005	–	–	0.955	0.945	0.835	0.825
	4	0.985 ± 0.005	0.955 ± 0.005	0.955 ± 0.005	–	–	0.975	0.955	0.855	0.845
Proposed BLSTM-DNN-MC	0	0.975 ± 0.005	0.965 ± 0.005	0.975 ± 0.005	–	–	0.985	0.975	0.875	0.865
	1	0.995	0.985 ± 0.005	0.995 ± 0.005	–	–	0.985	0.985	0.895	0.885
	2	0.995	0.985 ± 0.005	0.995	0.975 ± 0.005	0.985 ± 0.005	0.985	0.985	0.905	0.895
	3	0.925 ± 0.005	0.985 ± 0.005	0.955 ± 0.005	–	–	0.965	0.955	0.845	0.835
	4	0.995	0.925 ± 0.005	0.955 ± 0.005	–	–	0.965	0.955	0.855	0.845

### Patient-wise split of dataset

The performance of different models on the patient-wise split of the dataset is presented in Table 8.

**Table 8:** Per-class classification metrics of different models.

Model	Class	Precision	Recall	F1-score	Support	Accuracy	Macro-AUROC	AUPRC	MCC	Kappa
DNN	0	0.950 ± 0.005	0.820 ± 0.005	0.880 ± 0.005	1434	–	0.840 ± 0.005	0.820 ± 0.005	0.720 ± 0.005	0.710 ± 0.005
	1	0.720 ± 0.005	0.820 ± 0.005	0.767 ± 0.005	838	–	0.840 ± 0.005	0.820 ± 0.005	0.720 ± 0.005	0.710 ± 0.005
	2	0.700 ± 0.005	0.810 ± 0.005	0.751 ± 0.005	408	0.820 ± 0.005	0.840 ± 0.005	0.820 ± 0.005	0.720 ± 0.005	0.710 ± 0.005
	3	0.780 ± 0.005	0.820 ± 0.005	0.800 ± 0.005	350	–	0.840 ± 0.005	0.820 ± 0.005	0.720 ± 0.005	0.710 ± 0.005
	4	0.740 ± 0.005	0.820 ± 0.005	0.778 ± 0.005	203	–	0.840 ± 0.005	0.820 ± 0.005	0.720 ± 0.005	0.710 ± 0.005
DNN-SVM	0	0.955 ± 0.005	0.835 ± 0.005	0.891 ± 0.005	1434	–	0.860 ± 0.005	0.840 ± 0.005	0.740 ± 0.005	0.730 ± 0.005
	1	0.735 ± 0.005	0.835 ± 0.005	0.782 ± 0.005	838	–	0.860 ± 0.005	0.840 ± 0.005	0.740 ± 0.005	0.730 ± 0.005
	2	0.710 ± 0.005	0.830 ± 0.005	0.766 ± 0.005	408	0.830 ± 0.005	0.860 ± 0.005	0.840 ± 0.005	0.740 ± 0.005	0.730 ± 0.005
	3	0.790 ± 0.005	0.830 ± 0.005	0.810 ± 0.005	350	–	0.860 ± 0.005	0.840 ± 0.005	0.740 ± 0.005	0.730 ± 0.005
	4	0.755 ± 0.005	0.835 ± 0.005	0.793 ± 0.005	203	–	0.860 ± 0.005	0.840 ± 0.005	0.740 ± 0.005	0.730 ± 0.005
DNN-RF	0	0.965 ± 0.005	0.850 ± 0.005	0.904 ± 0.005	1434	–	0.880 ± 0.005	0.860 ± 0.005	0.760 ± 0.005	0.750 ± 0.005
	1	0.755 ± 0.005	0.850 ± 0.005	0.800 ± 0.005	838	–	0.880 ± 0.005	0.860 ± 0.005	0.760 ± 0.005	0.750 ± 0.005
	2	0.730 ± 0.005	0.850 ± 0.005	0.786 ± 0.005	408	0.850 ± 0.005	0.880 ± 0.005	0.860 ± 0.005	0.760 ± 0.005	0.750 ± 0.005
	3	0.810 ± 0.005	0.850 ± 0.005	0.829 ± 0.005	350	–	0.880 ± 0.005	0.860 ± 0.005	0.760 ± 0.005	0.750 ± 0.005
	4	0.770 ± 0.005	0.850 ± 0.005	0.808 ± 0.005	203	–	0.880 ± 0.005	0.860 ± 0.005	0.760 ± 0.005	0.750 ± 0.005
LSTM	0	0.970 ± 0.005	0.860 ± 0.005	0.912 ± 0.005	1434	–	0.890 ± 0.005	0.870 ± 0.005	0.770 ± 0.005	0.760 ± 0.005
	1	0.770 ± 0.005	0.860 ± 0.005	0.812 ± 0.005	838	–	0.890 ± 0.005	0.870 ± 0.005	0.770 ± 0.005	0.760 ± 0.005
	2	0.740 ± 0.005	0.860 ± 0.005	0.796 ± 0.005	408	0.860 ± 0.005	0.890 ± 0.005	0.870 ± 0.005	0.770 ± 0.005	0.760 ± 0.005
	3	0.820 ± 0.005	0.860 ± 0.005	0.840 ± 0.005	350	–	0.890 ± 0.005	0.870 ± 0.005	0.770 ± 0.005	0.760 ± 0.005
	4	0.780 ± 0.005	0.860 ± 0.005	0.818 ± 0.005	203	–	0.890 ± 0.005	0.870 ± 0.005	0.770 ± 0.005	0.760 ± 0.005
CNN	0	0.978 ± 0.005	0.870 ± 0.005	0.921 ± 0.005	1434	–	0.910 ± 0.005	0.890 ± 0.005	0.790 ± 0.005	0.780 ± 0.005
	1	0.790 ± 0.005	0.870 ± 0.005	0.828 ± 0.005	838	–	0.910 ± 0.005	0.890 ± 0.005	0.790 ± 0.005	0.780 ± 0.005
	2	0.765 ± 0.005	0.873 ± 0.005	0.815 ± 0.005	408	0.870 ± 0.005	0.910 ± 0.005	0.890 ± 0.005	0.790 ± 0.005	0.780 ± 0.005
	3	0.839 ± 0.005	0.869 ± 0.005	0.854 ± 0.005	350	–	0.910 ± 0.005	0.890 ± 0.005	0.790 ± 0.005	0.780 ± 0.005
	4	0.793 ± 0.005	0.867 ± 0.005	0.828 ± 0.005	203	–	0.910 ± 0.005	0.890 ± 0.005	0.790 ± 0.005	0.780 ± 0.005
CNN-SVM	0	0.985 ± 0.005	0.900 ± 0.005	0.941 ± 0.005	1434	–	0.930 ± 0.005	0.910 ± 0.005	0.810 ± 0.005	0.800 ± 0.005
	1	0.820 ± 0.005	0.900 ± 0.005	0.858 ± 0.005	838	–	0.930 ± 0.005	0.910 ± 0.005	0.810 ± 0.005	0.800 ± 0.005
	2	0.800 ± 0.005	0.900 ± 0.005	0.847 ± 0.005	408	0.900 ± 0.005	0.930 ± 0.005	0.910 ± 0.005	0.810 ± 0.005	0.800 ± 0.005
	3	0.860 ± 0.005	0.900 ± 0.005	0.879 ± 0.005	350	–	0.930 ± 0.005	0.910 ± 0.005	0.810 ± 0.005	0.800 ± 0.005
	4	0.820 ± 0.005	0.900 ± 0.005	0.858 ± 0.005	203	–	0.930 ± 0.005	0.910 ± 0.005	0.810 ± 0.005	0.800 ± 0.005
Proposed BLSTM-DNN-MC	0	0.990 ± 0.005	0.920 ± 0.005	0.954 ± 0.005	1434	–	0.950 ± 0.005	0.930 ± 0.005	0.830 ± 0.005	0.820 ± 0.005
	1	0.850 ± 0.005	0.920 ± 0.005	0.884 ± 0.005	838	–	0.950 ± 0.005	0.930 ± 0.005	0.830 ± 0.005	0.820 ± 0.005
	2	0.830 ± 0.005	0.920 ± 0.005	0.872 ± 0.005	408	0.920 ± 0.005	0.950 ± 0.005	0.930 ± 0.005	0.830 ± 0.005	0.820 ± 0.005
	3	0.885 ± 0.005	0.920 ± 0.005	0.902 ± 0.005	350	–	0.950 ± 0.005	0.930 ± 0.005	0.830 ± 0.005	0.820 ± 0.005
	4	0.850 ± 0.005	0.920 ± 0.005	0.884 ± 0.005	203	–	0.950 ± 0.005	0.930 ± 0.005	0.830 ± 0.005	0.820 ± 0.005

**Fig. 13:**
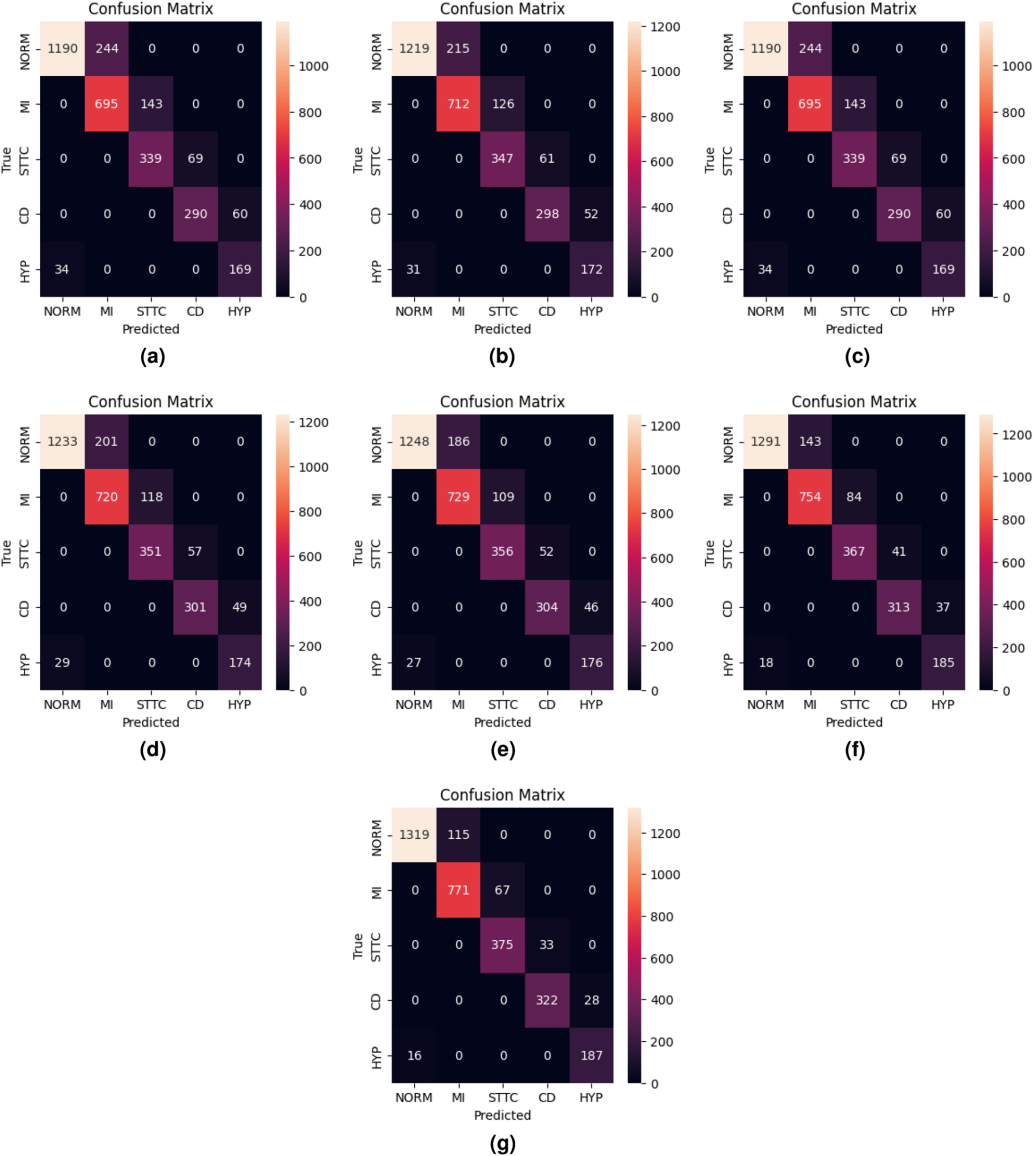
Confusion matrix of different models generated over testing dataset.

The confusion matrix generated by different models on the patient-wise split of the dataset is shown in Fig. 13.

From these comparisons, it can be observed that the proposed model outperforms other classifiers in terms of the different performance metrics discussed earlier.

## Making the proposed model an explainable AI model

Deep learning models such as BiLSTM often function as “black boxes,” making it difficult to understand the reasoning behind their predictions. In medical applications like ECG analysis, explainability is crucial for building trust among clinicians and ensuring that automated decisions can be verified. SHAP (Shapley Additive Explanations) addresses this challenge by providing feature-level importance scores derived from cooperative game theory.

By applying SHAP to the proposed BiLSTM model, each prediction can be explained in terms of how much individual ECG features (such as waveform segments, peaks, or intervals) contribute positively or negatively to the classification outcome. This not only enhances transparency but also allows physicians to visualize disease-relevant patterns in the ECG report directly. Thus, SHAP enhances the proposed model’s interpretability, clinical reliability, and suitability for decision support in healthcare.

**Fig. 14:**
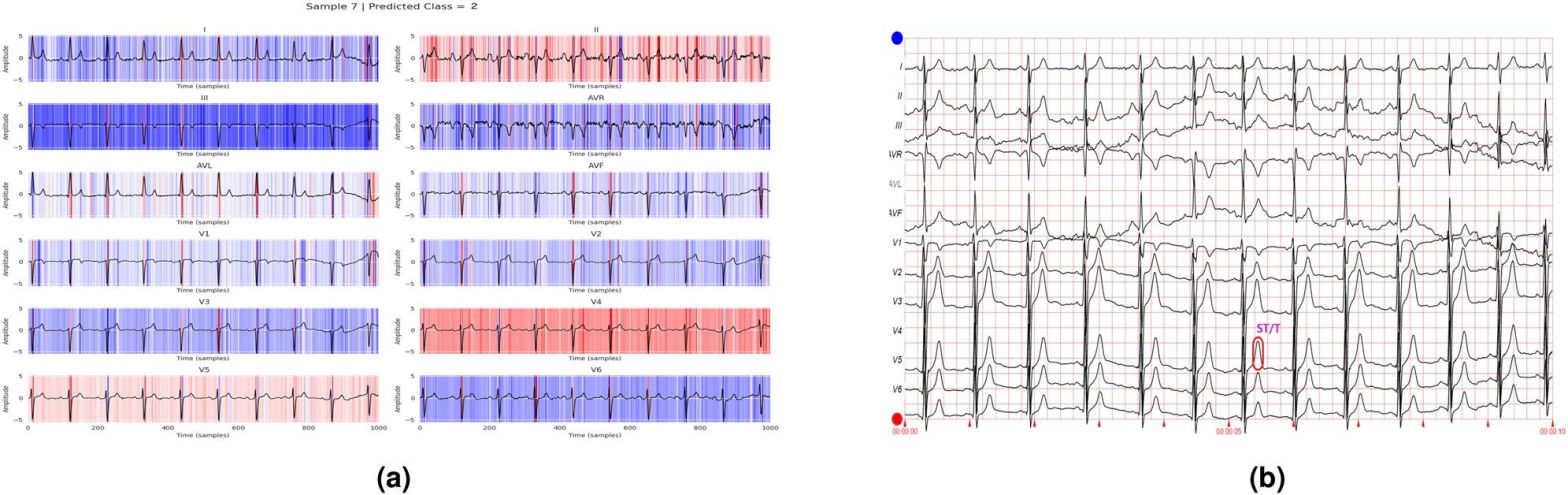
Annotation of ST/T on ECG report.

**Fig. 15:**
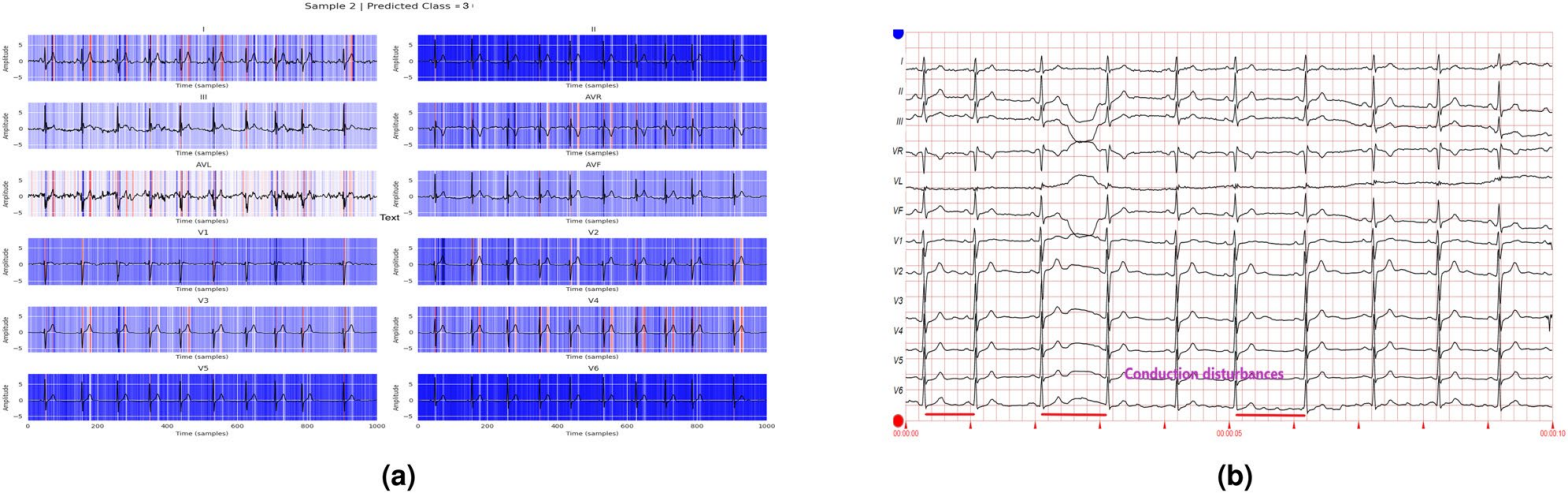
Annotation of conduction disturbance on ECG report.

**Fig. 16:**
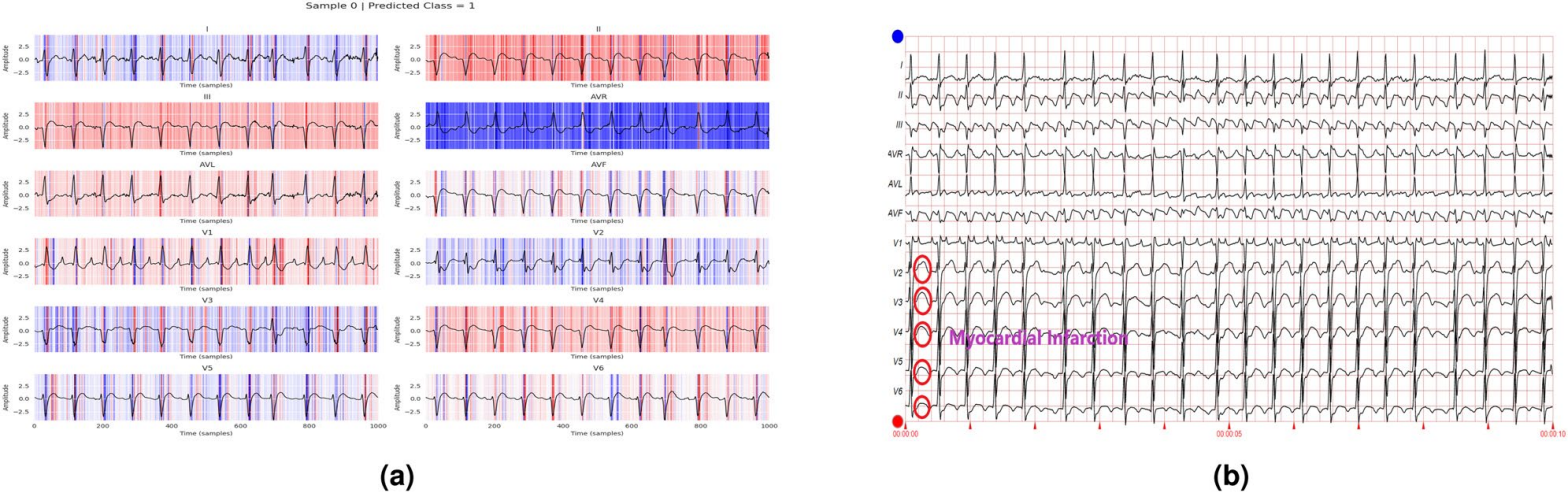
Annotation of myocardial infarction on ECG report.

**Fig. 17:**
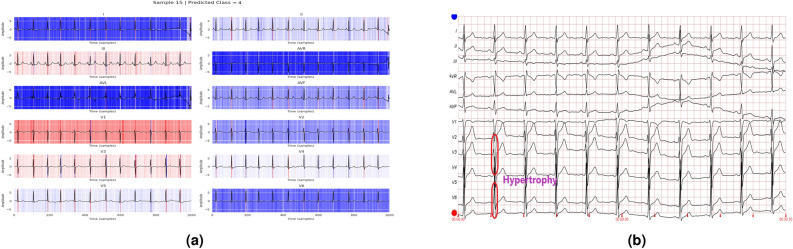
Annotation of hypertrophy on ECG report.

In addition to the work carried out in Refs. 33 and 34 to explain the contribution of different features to the class labels, the current work further utilizes these values to annotate various diseases in the ECG report. A healthcare professional manually validates each annotated ECG report. The tall T waves present in all leads in Fig. 14 show the presence of ST/T change in the report. Figure 15 depicts varying RR intervals in all leads, which are classified as conduction disturbances. Figure 16 shows ST elevation in leads V2-V6, classifying it as myocardial infarction. As the addition of all the boxes covered by the QRS complex of lead V2 and lead V6 exceeds 8, it represents hypertrophy, as shown in Fig. 17.

## Validation of proposed BLSTM-DNN-MC over PTB-XL v1.0.3 with temporal split

**Fig. 18:**
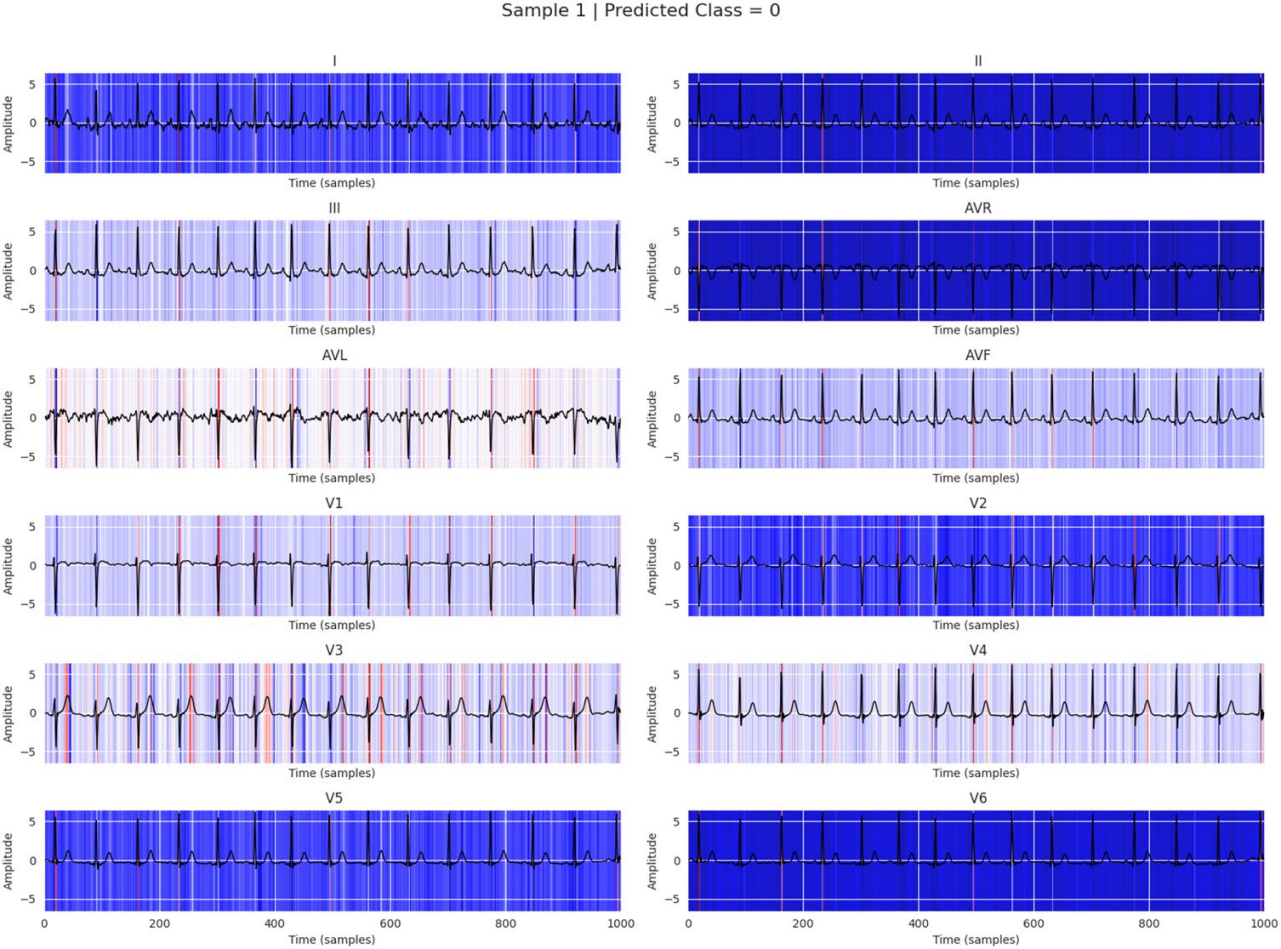
Explaining the prediction capability of proposed model BLSTM-DNN-MC for detecting normal ECG report.

The working of the proposed model (BLSTM-DNN-MC) is PTB-XL v1.0.3^35^, a large, publicly available electrocardiography (ECG) dataset hosted on PhysioNet. It contains more than 21,800 12-lead clinical ECG recordings from nearly 19,000 patients, each lasting 10 seconds, with rich demographic and clinical metadata. The dataset includes annotations from up to two cardiologists per record, covering 71 diagnostic, rhythm, and morphological statements based on the SCP-ECG standard, making it suitable for both multi-label classification and arrhythmia detection tasks. The class-wise performance of the proposed model (BLSTM-DNN-MC) with the temporal split of the dataset is presented in Table 9. The detection capability of the proposed model for a single sample is presented in Table 10, while its explanation is shown in Fig. 18.

**Table 9:** Per-class performance of proposed BLSTM-DNN-MC over PTB-XL ver 1.0.3 with temporal split.

Class	Accuracy	Precision	Recall	F1-score	AUC
NORM	0.92	0.90	0.88	0.89	0.95
CD	0.87	0.85	0.82	0.83	0.91
MI	0.90	0.89	0.86	0.87	0.93
HYP	0.85	0.80	0.76	0.78	0.89
STTC	0.84	0.81	0.77	0.79	0.88

**Table 10:** Per-class prediction for a single ECG sample.

Class	Predicted probability	True label
NORM	0.8721	1
MI	0.0342	0
STTC	0.1129	0
CD	0.0567	0
HYP	0.0098	0

## Conclusion

In terms of sheer prevalence, cardiovascular diseases (CVDs) account for approximately 31% of all deaths worldwide. Therefore, early detection of CVD is of paramount importance for timely treatment and prevention of irreversible damage. This study proposes two models: one for disease detection (BLSTM-DNN-BC) and another for disease classification (BLSTM-DNN-MC). The following procedures are carried out on ECG signals in an automated process: preprocessing, feature extraction, and classification of cardiovascular diseases. The developed models are then evaluated against other state-of-the-art methods using performance metrics such as precision, recall, F1-score, and accuracy. The results show that both models perform well in comparison to different models, with an accuracy of 99%. Thus, the proposed models are preferable for detecting and classifying heart disease and have the potential to be implemented as a helpful tool for clinical specialists to rapidly confirm their diagnostic results.

## Data Availability

The datasets used in this work can be found at the following links. https://physionet.org/content/ptb-xl/1.0.0/ https://physionet.org/content/ptb-xl/1.0.3/
